# A novel TFG variant of uncertain significance in amyotrophic lateral sclerosis: A case report and review of literature

**DOI:** 10.1016/j.amsu.2022.104840

**Published:** 2022-11-07

**Authors:** Bishal Dhakal, Sachin Sapkota, Aakriti Parajuli, Bibek Khadka, Binaya Subedi, Raju Paudel, Rohit Thapa, Sabin Rimal

**Affiliations:** a Nepalese Army Institute of Health and Sciences, College of Medicine, Kathmandu, Nepal; b Maulakalika Hospital Pvt. Ltd, Bharatpur-10, Chitwan, Nepal; c Chitwan Medical College, Bharatpur-10, Chitwan, Nepal

**Keywords:** Amyotrophic lateral sclerosis, Tropomyosin-receptor kinase fused gene, Endoplasmic reticulum, Exome sequencing, Neurodegeneration, ALS, Amyotrophic lateral sclerosis, MRI, Magnetic resonance imaging, TFG, Tropomyosin-receptor kinase fused gene

## Abstract

**Introduction:**

Amyotrophic lateral sclerosis is a neurodegenerative disease with wide variation of genetics associated with it. Among the different genes described, mutation in TFG is a rare finding in amyotrophic lateral sclerosis.

**Case presentation:**

A 35 years old right-handed male presenting with ipsilateral weakness was diagnosed with amyotrophic lateral sclerosis. He was found to have missense variant of TFG with uncertain significance on exome sequencing.

**Clinical discussion:**

The genetics involved in amyotrophic lateral sclerosis is ever-evolving. The identification of new TFG variant in this disease adds another evidence to the role of TFG in neurodegenerative disease.

**Conclusions:**

The finding of TFG variant of uncertain significance is a rare finding in amyotrophic lateral sclerosis. And with the identification of new TFG variant, it leads to further understanding of spectrum of TFG and its pathophysiology in amyotrophic lateral sclerosis.

## Highlights

• Amyotrophic lateral sclerosis is a neurodegenerative disease.
• Among the different genes described, mutation in TFG is a rare finding in amyotrophic lateral sclerosis.
• This expands the spectrum of TFG from oncogenesis to neurodegeneration in association with endoplasmic reticulum dysfunction.

## 1 Introduction

Amyotrophic lateral sclerosis (ALS) is a potentially incurable neurodegenerative disease. It is classically characterized by progressive muscle paralysis secondary to degenerations of motor neurons in cortex, brain-stem and spinal cord [[1], [2], [3], [4]]. The epidemiology of ALS in western world has been described as incidence and prevalence of 1.89 per 100,000/year and 5.2 per 100,000 respectively [3]. The different variants of ALS mentioned till date are limb-onset ALS, bulbar-onset, primary lateral sclerosis and progressive muscle atrophy [2]. Majority of the ALS cases are sporadic in origin. However, approximately 10% of the ALS cases account for familial origin [1,4].

Various genes have been implicated in ALS (as autosomal dominant or recessive) since the discovery of mutation in gene SOD1 (superoxide dismutase 1). They are responsible for both familial and sporadic ALS in either/or form. Some of the commonly described genes are SOD1, C9ORF72, TARDBP, FUS, TBK1 and PFN1 [[1], [2], [3], [4]]. The evolution of newer variations in genetic analysis for ALS is still on. This is a case report describing an atypical gene, TFG (tropomyosin-receptor kinase fused gene), implicated in a patient who was diagnosed as limb-onset ALS. As TFG gene is a rare finding in ALS, it is worth reporting it in our 35-years old patient diagnosed as ALS.

## 2 Case presentation

A 35-years-old right-handed Hindu male with no known comorbidities presented to neurology out-patient department (OPD) with weakness of ipsilateral (left) upper and lower limbs for three months. The weakness gradually progressed to left sided hemiparesis. He denied of facial deviation, loss of consciousness, drooling of saliva, abnormal body movements, any history of trauma lethargy and fatigue. There was no any relevant medical and surgical history. Family history was found to be insignificant.

On general examination, he was well-conscious and oriented to time, place and person. The vital parameters were stable. His higher mental functions were intact. The motor examination revealed power of 1/5–3/5 in medical research council (MRC) grading on left upper and lower limbs. The tone of left lower limbs was decreased as compared to the upper limbs. The lower limb had findings such as atrophy and fasciculations. The deep tendon reflexes were brisk on left upper limbs and lower limbs. There was foot drop in left side with positive Hoffman sign. The plantar reflex was upgoing on left side. There was no any sensory involvement. The bowel and bladder examination revealed no any abnormalities. The rest of the systemic examination was sound and intact. He was admitted for further evaluation.

**Fig. 1:**
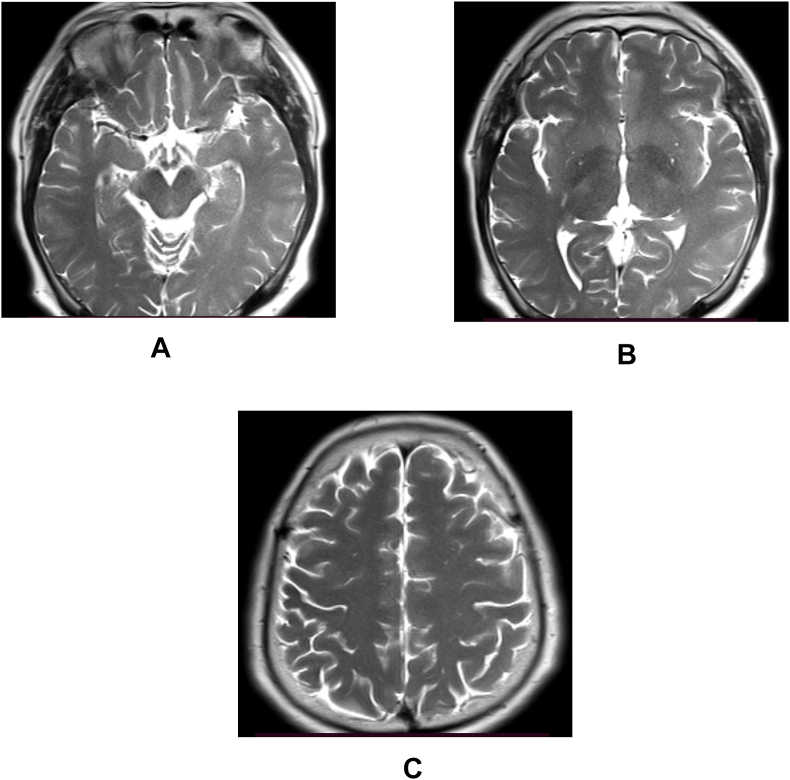
Axial T2-weighted MRI scan Legend (T2_a, T2_b and T2_c): Axial T2-weighted MRI showing normal scan with no any increased signal in corticospinal tracts.

The baseline investigations including complete blood count, renal and liver function tests, random blood sugar and electrolytes were within reference range. The magnetic resonance imaging (MRI) of the brain did not reveal any abnormalities as shown in Fig. 1. It revealed normal brain morphology with normal parenchymal signal intensity. The areas of internal capsule and brain stem had normal signal intensity. There was no any evidence of infarct or hemorrhages. Similarly, screening MRI of whole spine and brachial plexus was performed which did not have any significant findings except for mild degenerative changes in cervical and lumbar spine.

Later during the hospital stay, he developed tingling and burning sensation in his left medial forearm area. The nerve conduction study showed abnormal motor nerve conduction velocities (MNCV) in left median, ulnar and common peroneal nerve suggesting moderate axonal loss with demyelination. This was suggestive of motor neuron disease (MND).

The differentials considered were peripheral neuropathy, vitamin-B12 deficiency, thyroid disease, ischemic stroke, hereditary spastic paraplegia, myasthenia gravis and amyotrophic lateral sclerosis (ALS). The other possibilities were ruled out based on the history and clinical examination. He was then diagnosed ALS on the basis of Gold Coast criteria [[5], [6], [7]]. On exome sequencing [8], an atypical gene was isolated from gene sequencing as shown in Table 1.

**Table 1:** Atypical TFG gene in ALS.

Gene	Genomic location	Variant	Exon	Type	Zygosity	Condition/Phenotype group	Classification
TFG	Chr3:100463710	NM_001195478.1:c.755G > C; p.Gly252Ala	7	Missense	Heterozygous	Hereditary motor and sensory neuropathy; Okinawa type	Variant of Uncertain Significance (VUS)

The variant coverage statistics for TFG gene included reference allele coverage-G = 67 and alternate allele coverage-C = 57. The percentage target nucleotides covered included depth ≥20X, 93.97% coverage and quality threshold of 98.01%.

The treatment was started with edaravone with the initial cycle of 60 mg once daily for 14 days, followed by a 14-day drug free period. The subsequent cycles constituted 60 mg once daily for 10 days within a 14-day period, followed by a 14-day drug free period. Pregabalin and sertraline were also given as a supplementary treatment. He accepted the treatment and was discharged with the advice of follow-up for next edaravone cycle. He was followed-up during the edaravone cycle clinically. After completing four cycles of the edaravone treatment, he left the treatment. The clinical status was unchanged with some of the new symptoms like swallowing difficulty.

## 3 Discussion

Amyotrophic lateral sclerosis (ALS), in its familial and sporadic forms, has tremendous genetic importance. Till date, more than 120 genes have been implicated in ALS [9,10]. The most common genes associated in the etiopathogenesis of ALS are SOD1, TARDBP, FUS and C9ORF72 [9]. However, throughout the years many genes have evolved and linked to ALS and its variants. In contrast to the genes implicated in ALS, TFG (tropomyosin-receptor kinase fused gene) with variant of uncertain significance was an atypical finding in our case of ALS.

Although information regarding genetics in ALS is evolving, its variants with linked genes have been shown to have paramount of clinical importance [11]. The identification of genetics in ALS is crucial but not a simple process. Yet, it has importance in understanding the pathophysiological mechanisms of the disease [12]. The etiopathogenesis of ALS is complex and still debatable. However, majority of cases show TDP-43 positive ubiquitinated cytoplasmic inclusions in pathology [10].

Tropomyosin-receptor kinase fused gene (TFG) was originally described as fusion protein associated with the formation of oncogenic products in multiple cancers like thyroid carcinomas, lymphomas and soft tissue tumors [13,14]. TFG serves an important role in oncogenesis, cell apoptosis and cellular proliferation. Its main function has been described as a role in protein secretion. In the molecular level, it assembles with Sec16, a protein in endoplasmic reticulum (ER), thus associated with ER function for intracellular protein secretion [15,16].

With the recent advances, ER dysfunction is being linked with neurodegeneration. Similarly, ER stress is also identified in the etiopathogenesis of ALS. TFG is also an inhibitory regulator of ubiquitin-proteasome system (UPS). And this is responsible for unfolded protein response (UPR), which finally results in cell death [15,17]. This suggests that the mutation in TFG results in disruption of intracellular protein homeostasis in nervous system too [[18], [19], [20]]. And this leads to the neurogenerative diseases by affecting neuronal axons. The association of TFG with nervous system diseases has been increasing recently. It has been linked with hereditary motor and sensory neuropathy with proximal dominant involvement (HMSN-P) [21], Charcot-Marie-Tooth disease type 2 (CMT2) [22] and hereditary spastic paraplegia (SPG57) [23]. A recent report of 2022 has also shown the role of a novel mutation in TFG [novel variant of TFG (c.1148 G > A, p. Arg383His)] in the pathogenesis of α-synucleinopathy (Parkinson's disease) or TDP-43 proteinopathy (ALS) [24].

Though TFG mutation has been linked with neurodegenerative diseases, its association with ALS has not been shown yet except for the single report as mentioned above. In regard to our case, heterozygous missense variant was detected in TFG gene (c.755G > C; p. Gly252Ala) which was unique and not reported before in the existing literature. The In Silico prediction tool predicted the identified variant to be damaging by DANN, FATHMM-MKL, SIFT and Mutation Taster. It was novel as per gnomAD database and had been not previously reported in ClinVar database. Hence this variant was classified as a Variant of Uncertain Significance (VUS). And this was not reported in ALS case previously.

ALS is a fatal disease with only progressive nature from the appearance of signs and symptoms. There are two food and drug administration (FDA) approved drug for the treatment of ALS: Riluzole and Edaravone. These both have role only in slowing the progression of the disease [10]. And the prognosis of this disease is still variable and difficult to predict. As all the cases of ALS are progressive and culminate with death, supportive care takes its place in the later course of the disease [1,2,10]. However, with the identification of various genes and their association in pathophysiological mechanism of ALS, novel therapeutics targeting those genes and molecular channels are being developed and under research at current time.

As TFG was a unique finding associated with our ALS case, it has been reported and described here with the relevant literature. Some of the limitations encountered during the study were unable to take detail family history with pedigree charts and unable to do follow up with the patient at every time he visited the hospital.

## 4 Conclusions

In ever-evolving genetics of ALS, finding of TFG variant of uncertain significance is a rare finding. This expands the spectrum of TFG from oncogenesis to neurodegeneration in association with ER dysfunction. And it paves the way for potential new therapeutics in search for the cure of ALS.

## Author agreement statement

We the undersigned declare that this manuscript is original, has not been published before and is not currently being considered for publication elsewhere.

We confirm that the manuscript has been read and approved by all named authors and that there are no other persons who satisfied the criteria for authorship but are not listed. We further confirm that the order of authors listed in the manuscript has been approved by all of us.

We understand that the Corresponding Author is the sole contact for the Editorial process. He/she is responsible for communicating with the other authors about progress, submissions of revisions and final approval of proofs.

## Ethical approval

This is a case report, therefore, it did not require ethical approval from ethics committee.

## Funding

The study did not receive any grant from funding agencies in the public, commercial or not-for-profit sectors.

## Author contribution

1. Bishal Dhakal.

2. Sachin Sapkota.

3. Aakriti Parajuli.

4. Bibek Khadka.

5. Binaya Subedi.

6. Raju Paudel.

7. Rohit Thapa.

8. Sabin Rimal.

## Trail registry number

Not applicable.

## Guarantor

Bishal Dhakal, Nepalese Army Institute of Health and Sciences, 44600 Kathmandu, Nepal. Email: swarnimdhakal@gmail.com, Phone: +977 9846491651.

## Consent

Written informed consent was obtained from the patient for publication of this case report and accompanying images. A copy of the written consent is available for review by the editor-in-chief of this journal on request.

## Declaration of competing interest

The authors report no conflicts of interest.
